# Differences in the Flexibility of Switching Learning Strategies and CREB Phosphorylation Levels in Prefrontal Cortex, Dorsal Striatum and Hippocampus in Two Inbred Strains of Mice

**DOI:** 10.3389/fnbeh.2016.00176

**Published:** 2016-09-16

**Authors:** Woo-Hyun Cho, Jung-Soo Han

**Affiliations:** Department of Biological Sciences, Konkuk UniversitySeoul, South Korea

**Keywords:** cAMP response element-binding protein, strategy-switching, place learning, cued learning, hippocampus, striatum, prefrontal cortex, water maze

## Abstract

Flexibility in using different learning strategies was assessed in two different inbred strains of mice, the C57BL/6 and DBA/2 strains. Mice were trained sequentially in two different Morris water maze protocols that tested their ability to switch their learning strategy to complete a new task after first being trained in a different task. Training consisted either of visible platform trials (cued training) followed by subsequent hidden platform trials (place training) or the reverse sequence (place training followed by cued training). Both strains of mice showed equivalent performance in the type of training (cued or place) that they received first. However, C57BL/6 mice showed significantly better performances than DBA/2 mice following the switch in training protocols, irrespective of the order of training. After completion of the switched training session, levels of cAMP response element-binding protein (CREB) and phosphorylated CREB (pCREB) were measured in the hippocampus, striatum and prefrontal cortex of the mice. Prefrontal cortical and hippocampal pCREB levels differed by strain, with higher levels found in C57BL/6 mice than in DBA/2 mice. No strain differences were observed in the medial or lateral region of the dorsal striatum. These findings indicate that the engagement (i.e., CREB signaling) of relevant neural structures may vary by the specific demands of the learning strategy, and this is closely tied to differences in the flexibility of C57BL/6 and DBA/2 mice to switch their learning strategies when given a new task.

## Introduction

Different neural systems can be recruited during learning, depending on whether place/spatial (place) or cued/response (cued) strategies are used to navigate a complex environment (McDonald and White, 1994; Packard and McGaugh, 1996; Hartley et al., 2003). Specifically, in the water maze task, a place learning strategy is an allocentric navigation strategy that relies on spatial information and requires hippocampal function. In contrast, a cued learning strategy is an egocentric navigation strategy that requires instrumental learning and depends on the striatal system. Consistent with these roles, functionally disrupting or lesioning the hippocampus impairs place learning in rat and monkeys (Packard and McGaugh, 1996; Lavenex et al., 2006) and damage or inactivation of the striatum impairs cued learning (McDonald and White, 1994; Packard and McGaugh, 1996). In addition, recent studies have revealed interactions between the two systems and have implicated the prefrontal cortex in switching of learning strategy (Arias et al., 2014).

Both C57BL/6 and DBA/2 mice are frequently used as the background strains of genetically modified mice in studies that are focused on the identification of molecular mechanisms critical for learning and memory function (Brooks et al., 2005; Mishina and Sakimura, 2007). The performance of C57BL/6 and DBA/2 mice has been studied extensively in learning and memory tasks, with significant differences reported. C57BL/6 mice perform significantly better than DBA/2 mice in hippocampal-dependent tasks, such as place learning in the Morris water maze test or contextual fear conditioning (Paylor et al., 1993, 1994). In addition to these behavioral differences, strain differences in the functional neuroanatomical and neurochemical systems that support these behaviors have also been reported. For example, DBA/2 mice show deficient maintenance of long-term potentiation in the dentate gyrus and lower hippocampal expression levels of protein kinase C (PKC) activity relative to C57BL/6 mice (Matsuyama et al., 1997; Bampton et al., 1999). Consequently, C57BL/6 mice have been used preferentially as the genetic background for conditional gene targeting (Mishina and Sakimura, 2007) due to their superior performance in learning and memory tasks. However, despite hippocampal differences between two inbred mice, other studies have reported no differences in performance between C57BL/6 and DBA/2 mice on hippocampus-dependent tasks (Ammassari-Teule and Caprioli, 1985; Owen et al., 1997; Brooks et al., 2005; Middei et al., 2007).

Given these somewhat disparate findings and the importance of identifying the best background strains to support genetic studies of learning and memory, we recently investigated specific learning strategies in these two strains and the underlying molecular signaling in hippocampus or striatum. Specifically, hippocampal phosphorylated cAMP response element-binding protein (pCREB) was evaluated following either place or cued training in the water maze. Both strains performed similarly in both place and cued tasks. While no differences in hippocampal pCREB were observed between the C57BL/6 mice and DBA/2 mice after cued learning, hippocampal pCREB was higher in the C57BL/6 mice than DBA/2 mice after place learning (Sung et al., 2008). In a companion study, we assessed behavioral strategy preferences of C57BL/6 and DBA/2 mice in a redundant place/cue version of the water maze task adapted from McDonald and White (1994). In this task, mice received redundant cued and place training to a single location in a water maze. These strategies were then placed in competition, with the mice required to choose between the original spatial location of a hidden platform (a place strategy) and a visible platform located in a new location (a cued strategy). In this test, C57BL/6 mice visited the place location first before escaping to the visible platform significantly more often than DBA/2 mice. These results demonstrated a strong preference of C57BL/6 mice for using a place learning strategy in a task when both place and cued strategies are available. On the other hand, the differences between the two strains might also reflect a superior ability of C57BL/6 mice to switch their learning strategies.

To investigate this issue further, the present study directly examined strain differences in the ability to switch learning strategies in the Morris water maze and in CREB and pCREB levels in the hippocampus, striatum, and prefrontal cortex, which were used as indices of the engagement of these areas in response to specific training sequences (Colombo et al., 2003; Dalley et al., 2004; Floresco et al., 2008).

## Materials and Methods

### Animals

Thirty-five male C57BL/6 and 35 male DBA/2 mice (3 months old, SPF) were obtained from Charles River Co. (Gapeung, South Korea) at the beginning of the experiments. Mice were housed in groups of 4 per cage in a temperature- and humidity-controlled room on a 12 h light/dark cycle (lights on 07:00–19:00). Food and water were available *ad libitum*. All testing was performed during the light cycle. The Institutional Animal Care and Use Committee of Konkuk University approved all protocols described in this study.

### Apparatus

The water maze consisted of a circular tank (1.83 m diameter and 0.58 m height) with an escape platform (20 cm diameter) centered in one of the four maze quadrants. Water (27°C) was made opaque by the addition of nontoxic white paint. The visible escape platform was raised 2 cm above the water surface for cued training. The hidden platform was located 0.5 cm beneath the surface for place training. The maze was surrounded by white curtains (50 cm from the pool periphery), on which black felt patterns were affixed to provide three black extramaze (spatial) cues. These spatial cues were not placed directly to the hidden platform, such that it was not possible to discern from above the water, which cue was associated with the platform for the possibility of escape. Data were recorded using an HVS Image tracking system (Hampton, UK).

### Behavioral Training Procedure

Cued training: mice received four trials/day (with 10 min intertrial intervals (ITIs), maximum trial duration of 60 s, with 20 s on the platform at the end of each trial) in which a visible platform was moved to different locations in the pool between trials. Blank white curtains were drawn around the pool during cue training to occlude extramaze cues.

Place training: mice received four trials/day (10 min ITI, maximum trial duration of 60 s, with 20 s on the platform at the end of each trial), with each trial beginning at one of four located positions at the perimeter of the maze. The location of the platform remained constant across all training trials. Mice were placed into the water facing the wall and were allowed to swim for a maximum of 60 s. The trial ended when a mouse climbed onto the available platform or after the 60 s interval had elapsed. If a mouse did not locate the platform during a trial, it was placed on the platform by the experimenter. Mice were left on the platform for 20 s and then moved to a holding cage for a 10 min ITI.

Learning strategy-switching task: one cohort of C57BL/6 and DBA/2 mice received cued training for 4 days, followed by place training for 4 days (cued training → place training). Conversely, the other cohort of mice was trained in the opposite order (place training → cued training). Thirty minutes after the last training trial on the eighth day in both protocols, all mice were sacrificed.

### Western Blots

The brains were removed from the mice and the entire hippocampus was dissected bilaterally on the ice-cold metal stage. The dorsal striatum was dissected as described in detail elsewhere (Gallagher et al., 1990). For preparation of the prefrontal cortex, the brains were placed with the ventral side facing the metal plate on ice. Using a blade, the brains were cut between olfactory bulb and striatum, and the prefrontal cortex was sectioned by a dissection pin. The hippocampus, striatum, and prefrontal cortex were rapidly dissected and frozen at −80°C until further processing. To extract proteins for the analysis of CREB and pCREB, individual tissue samples were weighed and then homogenized in 5 vol of ice-cold buffer containing 20 mM Tris (pH 7.5), 5% glycerol, 1.5 mM EDTA, 40 mM KCl, 0.5 mM dithiothreitol, and protease inhibitors (No. 539131, Calbiochem). Homogenates were centrifuged at 14,000 rpm for 1 h at 4°C. The supernatant was removed from each sample, and an aliquot was taken for determination of total protein concentration using the Bradford reagent. The proteins were then separated by sodium dodecyl sulfate (SDS)-polyacrylamide gel electrophoresis and transferred to a Polyvinylidene difluoride (PVDF) membrane. Membranes were incubated with primary antibodies (Ab) against pCREB, phosphorylated on serine-133 (Millipore, #06-519) and total CREB (Cell Signaling, #9197). Following primary incubation, blots were incubated with an HRP-conjugated secondary Ab (Cell Signaling, #7074). Blots were visualized using an ECL system and Hyperfilm (Amersham). The relative expression levels of pCREB and CREB were determined by densitometry and normalized to β-actin (Sigma), a ubiquitous cytoskeletal protein.

### Statistical Analyses

Escape latencies during training were analyzed using a repeated measures two-factor analysis of variance (ANOVA; strain × trial session [day]) to evaluate learning acquisition in the place and cued training tasks. Three or two-factor ANOVA was conducted with levels of CREB and pCREB as dependent variables. Independent variables were either learning strategy-switching order (cued → place vs. place → cued), the two mouse strains (C57BL/6 vs. DBA/2), or the dorsal striatal region examined (medial vs. lateral). It was followed by Bonferroni’s *post hoc* comparisons. A *p*-value < 0.05 was considered significant. All data are expressed as the mean ± SEM.

## Results

### C57BL/6 Mice Showed Superior Performance in the Learning Strategy-Switching Task Compared With DBA/2 Mice

**Figure 1:**
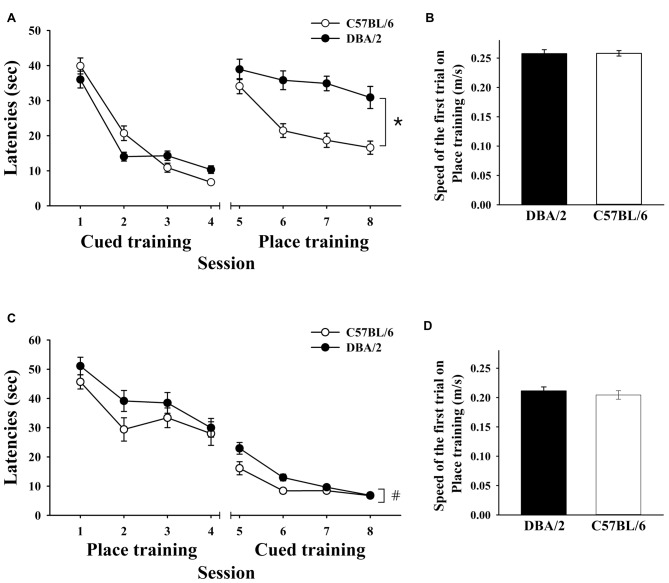
**Performance of C57BL/6 and DBA/2 mice in the learning strategy-switching task. (A)** Behavioral data from C57BL/6 and DBA/2 mice (*n* = 16/strain) that received cued training for 4 days and then place training for 4 days. After switching to place training, C57BL/6 mice perform significantly better than DBA/2 mice (*). **(B,D)** Swimming speeds of the two strains of mice do not differ in the first trial of either the cued or place training. **(C)** Behavioral data from the second cohort of C57BL/6 and DBA/2 mice (*n* = 16/strain) that received place training first and then cued training. After switching to cued training from place training, C57BL/6 mice again perform significantly better than DBA/2 mice (#).

Male mice were trained to escape to a visible platform for 4 days and then a hidden platform for 4 days. In the first task, C57BL/6 and DBA/2 mice exhibited similar performances (Figure 1A). Both C57BL/6 and DBA/2 mice improved over the course of the cued training, as measured by reduced escape latencies (*F*_(3,90)_ = 152.05, *p* < 0.001). There was no significant overall effect of strain on performance (*F*_(1,30)_ = 0.38, *p* = 0.54) but a significant strain-by-session interaction effect was detected (*F*_(3,90)_ = 6.00, *p* < 0.001), reflecting different learning rates between the strains. In the place training that followed, the escape latencies of C57BL/6 mice, but not the DBA/2 mice, dramatically decreased over the course of training (Figure 1A). Statistical analyses revealed that the main effects of session (*F*_(3,90)_ = 11.22, *p* < 0.001) and strain (*F*_(1,30)_ = 43.85, *p* < 0.001) were significant, but strain-by-session interaction effects were not (*F*_(3,90)_ = 2.51, *p* = 0.064). The two strains of mice showed no differences in swimming speeds during these training tasks (*t*_(30)_ = 0.076, *p* = 0.940; Figure 1B).

Another cohort of mice was trained to escape to a hidden platform for 4 days and then a visible platform for 4 days. C57BL/6 and DBA/2 mice exhibited similar learning rates during the place training, as indicated by progressively decreased latencies to find the hidden platform. Both C57BL/6 and DBA/2 strains improved over the course of place training (*F*_(3,90)_ = 17.21, *p* < 0.001). No effect of strain (*F*_(1,30)_ = 2.62, *p* = 0.12) or strain-by-session interaction (*F*_(3,90)_ = 0.65, *p* = 0.59) on behavioral performance was found. Interestingly, in the cued learning trials that followed, C57BL/6 mice performed better than DBA/2 mice. Statistical analysis of the performances of the two strains of mice during the switched training task revealed that C57BL/6 and DBA/2 mice improved performance over the course of the cued training (*F*_(3,90)_ = 51.89, *p* < 0.001). A significant main effect of strain was found (*F*_(1,30)_ = 6.85, *p* = 0.014; Figure 1C) and also a significant strain-by-session interaction effect (*F*_(3,90)_ = 3.96, *p* = 0.011), reflecting different learning rates. The two strains of mice showed no differences in swimming speeds during this training (*t*_(30)_ = 0.694, *p* = 0.493; Figure 1D).

### Hippocampal pCREB Levels of C57BL/6 Mice Were Significantly Increased After the Switch in Training Tasks

**Figure 2:**
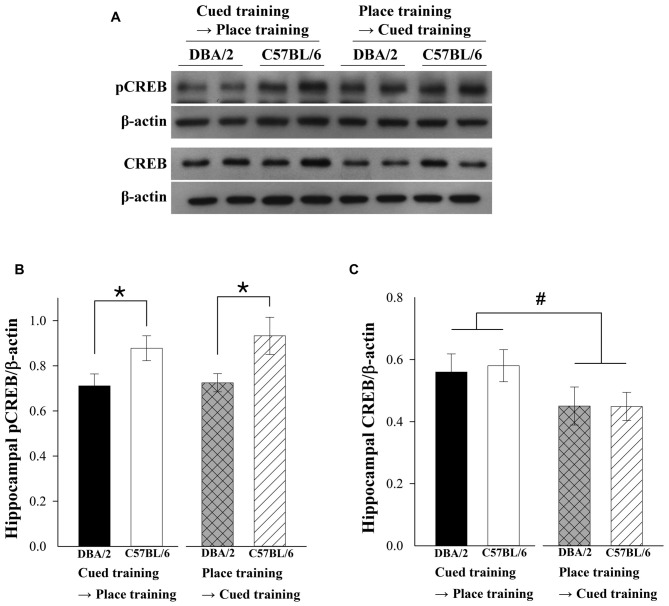
**Hippocampal levels of phosphorylated cAMP response element-binding protein (pCREB) and CREB in the C57BL/6 and DBA/2 mice following the learning strategy-switching task.** Representative immunoblots **(A)** of hippocampal pCREB and CREB from C57BL/6 and DBA/2 mice following the completion of the switched task and their quantification **(B,C)**. Hippocampal pCREB levels of C57BL/6 are higher than those of DBA/2 mice in both learning strategy-switching tasks (*, **B**). Hippocampal CREB levels are lower in both C57BL/6 and DBA/2 mice receiving cued-to-place training compared to those receiving place-to-cued training (#, **C**).

After the last training trial, the two strains of mice were sacrificed and their hippocampal pCREB and CREB levels were measured. Figure 2A shows representative immunoblots of hippocampal pCREB, CREB, and actin. Two-way ANOVAs were conducted to examine the effects of strain (C57BL/6 vs. DBA/2) and learning strategy-switching order (cued → place vs. place → cued) on pCREB and CREB levels. In the pCREB analysis (Figure 2B), a significant effect of strain (*F*_(1,36)_ = 9.83, *p* < 0.01) was evident. The hippocampal pCREB levels were increased after both learning strategy-switching tasks in C57BL/6 compared to DBA/2 mice. However, no significant learning strategy-switching order (*F*_(1,36)_ = 0.33, *p* = 0.57) or learning strategy-switching order-by-strain interaction effect (*F*_(1,36)_ = 0.12, *p* = 0.73) was found. A significant effect of learning strategy-switching order (*F*_(1,28)_ = 4.213, *p* = 0.05) was evident in the CREB analysis (Figure 2C). The hippocampal CREB levels were higher in both C57BL/6 and DBA/2 mice receiving place-to-cued training compared to those receiving cued-to-place training. However, no significant strain effect or learning strategy-switching order-by-strain interaction effect (*F*_(1,28)_ = 0.26, *p* = 0.87; *F*_(1,28)_ = 0.35, *p* = 0.85) was observed.

### Levels of pCREB and CREB in the Medial and Lateral Regions of the Dorsal Striatum of C57BL/6 and DBA/2 Mice After the Learning Strategy-Switching Task

**Figure 3:**
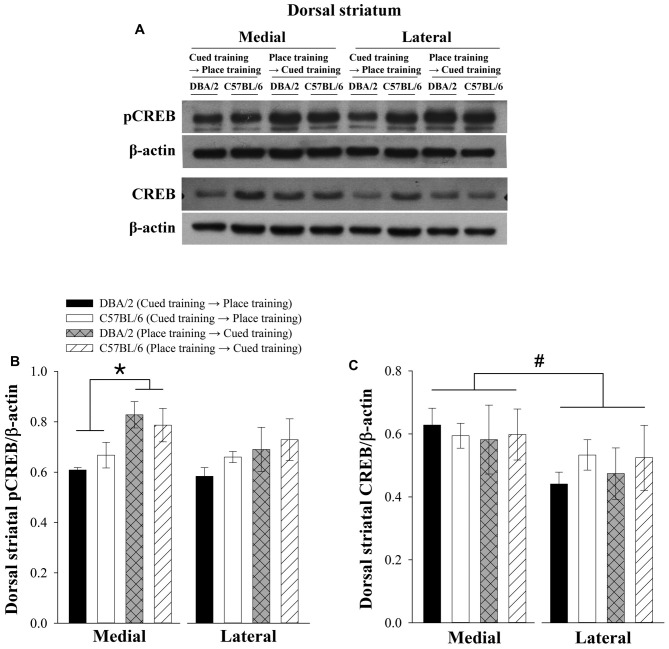
**Levels of pCREB and CREB in the lateral and medial regions of the dorsal striatum following the learning strategy-switching task.** Representative immunoblots **(A)** of dorsal striatal pCREB and CREB from C57BL/6 and DBA/2 mice after the completion of the switched training task and their quantification **(B,C)**. Levels of pCREB in the medial region of the dorsal striatum are lower in C57BL/6 and DBA/2 mice receiving cued-to-place training compared to those receiving place-to-cued training (*, **B**). Regional differences are not observed in pCREB levels but are detected in CREB levels (#, **C**).

Previous studies have indicated that the medial and lateral regions of the dorsal striatum might have different functions (Brown and Robbins, 1989; Eagle et al., 1999). The medial region processes spatial information while the lateral region handles response information. Therefore, pCREB and CREB levels in the medial and lateral regions of the dorsal striatum were measured. Figure 3A shows representative immunoblots of dorsal striatal pCREB, CREB, and actin. Three-way ANOVAs were conducted to analyze the expression levels of pCREB or CREB as dependent variables. The pCREB levels of dorsomedial striatum were lower in C57BL/6 and DBA/2 mice receiving cued-to-place training compared to those receiving place-to-cued training. Independent variables were the learning strategy-switching order (cued → place training vs. place → cued), strain (C57BL/6 vs. DBA/2), and dorsal striatal region (medial vs. lateral). Only levels of pCREB significantly differed with learning strategy-switching order (*F*_(1,40)_ = 13.98, *p* < 0.01, Figure 3B). In addition, a significant effect of striatal region on the levels of CREB was observed (*F*_(1,40)_ = 5.468, *p* < 0.05, Figure 3C).

### Strain Differences in pCREB Levels in Prefrontal Cortex Were Observed After the Learning Strategy-Switching Task from Cued to Place Training

**Figure 4:**
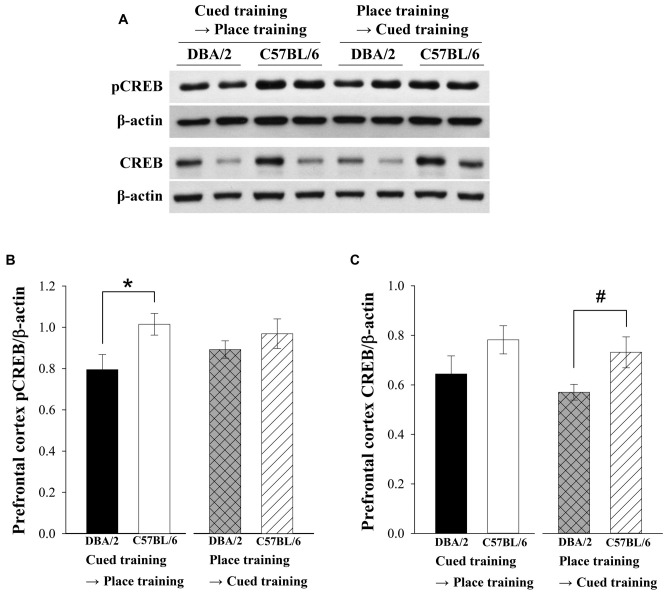
**Levels of pCREB and CREB in the prefrontal cortex of the C57BL/6 and DBA/2 mice following the learning strategy-switching task.** Representative immunoblots **(A)** of prefrontal cortex pCREB and CREB in C57BL/6 and DBA/2 mice **(A)** and their quantification **(B,C)**. After the cued-to-place training protocol, pCREB levels in the prefrontal cortex of C57BL/6 mice are significantly higher than those in DBA/2 mice (*, **B**). CREB levels of C57BL/6 are higher than those of DBA/2 mice following place-to-cued training (#, **C**).

Recent studies have reported that prefrontal cortex is involved in strategy switching and executive functions (Dalley et al., 2004; Floresco et al., 2008). Therefore, pCREB and CREB levels of prefrontal cortex were measured in C57BL/6 and DBA/2 mice after finishing the switched training trials. Two-way ANOVAs were conducted to analyze pCREB and CREB levels in prefrontal cortex as a dependent variable. The pCREB levels of prefrontal cortex in C57BL/6 were higher than DBA/2 mice receiving cued-to-place training. Independent variables were the learning strategy-switching order (cued → place vs. place → cued) and mouse strain (C57BL/6 vs. DBA/2). The effect of strain on pCREB levels (*F*_(1,28)_ = 5.83, *p* < 0.05) was significant (Figures 4A,B), but the effect of learning strategy-switching order (*F*_(1,28)_ = 0.18, *p* = 0.68) and the interaction effects of strain-by-learning strategy-switching order (*F*_(1,28)_ = 1.37, *p* = 0.25) were not. Similar to the results for pCREB, only the effect of strain on CREB levels was significant (*F*_(1,28)_ = 6.65, *p* < 0.05; Figure 4C).The CREB levels of C57BL/6 mice were higher than DBA/2 mice following place-to-cued training. That is, expression levels of CREB in the prefrontal cortex were higher in C57BL/6 mice than in DBA/2 mice.

## Discussion

The present study examined the flexibility in switching learning strategies in a water maze in two inbred mice strains, C57BL/6 and DBA/2 mice, which received either cued training followed by place training or training in the reverse order. C57BL/6 and DBA/2 mice showed similar performances when either cued training or place training was the initial training protocol received. Consistent with the present results, a previous study reported no behavioral differences following either cued or place training between C57BL/6 and DBA/2 mice that had received no prior exposure to the maze or any prior training (Sung et al., 2008). DBA/2 mice, however, performed worse in the switched training task, regardless of the training order, suggesting that C57BL/6 mice have superior ability of switching from one learning strategy to another.

Interestingly, in the present study, large behavioral differences between C57BL/6 and DBA/2 mice were observed when place training followed cued training. The poor performance of DBA/2 mice in this task could be interpreted to be the result of hippocampal dysfunction. For example, measures of synaptic plasticity, such as long-term potentiation, are more robust in C57BL/6 mice than DBA/2 mice (Matsuyama et al., 1997; Nguyen, 2006). Furthermore, basal levels of hippocampal membrane PKC activity, which plays key roles in learning and memory processing, are higher in C57BL/6 mice (Wehner et al., 1990; Bowers et al., 1995). However, despite these hippocampal differences, several reports including ours have shown that C57BL/6 and DBA/2 mice perform similarly when trained in the hidden platform of the Morris water maze for the first time (Owen et al., 1997; Middei et al., 2007), which is not compatible with hippocampal dysfunction.

No differences between two strains in place learning could be explained by the finding that DBA/2 mice could locate a hidden platform by relying on vestibular signals (Middei et al., 2007). In this experiment, the procedure that disturbs vestibular information processing, rotating before each training trial, disrupted DBA/2 performance without altering C57BL/6 performance, suggesting that DBA mice rely on path integration to localize the hidden platform without engaging their hippocampus. If so, DBA/2 hippocampus would not be used in the navigation, and in turn, place learning-induced changes would not be observed. In fact, less mossy fiber synaptogenesis or activation of CREB signaling after place learning was reported in DBA/2 mice relative to C57BL/6 mice (Middei et al., 2007; Sung et al., 2008). In the same context, it is possible that performance of DBA mice is much impaired in the cued-place switch than in the place-cued switch relative to those of C57BL/6 mice, because switching from a cued strategy to a path integration strategy is more difficult than switching from a cued to a place strategy that C57BL/6 mice might do.

However, C57BL/6 mice have been displayed higher freezing than DBA/2 mice in the hippocampal-dependent context or trace fear conditioning requiring no navigation (Nguyen et al., 2000; Hwang et al., 2010). In our companion study (2010) examined hippocampal pCREB levels of C57BL/6 and DBA/2 mice received the trace fear conditioning (paired vs. unpaired), hippocampal p-CREB levels of C57BL/6 mice with paired were higher than those of DBA/2 mice, but no differences were observed between two mice strains with unpaired (Hwang et al., 2010). We also measured CREB and pCREB levels in the hippocampus, striatum, and prefrontal cortex of two strain mice which were placed at the location of water maze training for 4 days. No significant differences between C57BL/6 and DBA/2 mice were observed in pCREB levels in the brain regions examined (unpublished data). Thus, because pCREB alteration is closely tied with training or learning, it is likely that the hippocampus of DBA/2 mice is functionally impaired relative to those of C57BL/6 mice.

Intriguingly, in the present experiment, the same p-CREB hippocampal activation was detected following cued training in the place-cued switch or place training in the cued-place switch. Specifically, relative to DBA/2 mice, C57BL/6 mice showed increased p-CREB levels in the hippocampus following the switched training, regardless of switching order. These results would be explained by the recent study showing that the hippocampus play vital roles in the reversal or set-shift learning related to flexibility, along with prefrontal cortex (Rubin et al., 2014; Malá et al., 2015) and could also be interpreted at the light of data reporting that C57BL/ mice might process hippocampal-dependent contextual information (Pignataro et al., 2013) better than DBA/2 mice.

Behavioral differences between the two strains of mice were also observed when they received sequential place-to-cued training in the water maze. As previously observed, DBA/2 mice were able to improve their performance in cued training despite exhibiting longer latencies to find the visible platform compared to C57BL/6 mice. Given that damage to the dorsal striatum adversely affects cue or response learning, a dysfunction in this region might underlie the poorer performance of DBA/2 mice in the cued task. However, no prior study has examined functional or anatomical differences in the dorsal striatum between these two inbred mouse strains. In a learning situation requiring the processing of cued information, the dorsal striatum was markedly more activated in DBA/2 mice than in C57BL/6 mice (Passino et al., 2002). Therefore, dysfunction of neither the hippocampus nor the dorsal striatum explains the poor performance of DBA/2 mice in the learning strategy-switching task.

In order to determine which brain regions might be involved in the superior performance of C57BL/6 mice over DBA/2 mice in the learning strategy-switching task, we measured the expression levels of CREB and pCREB proteins in the hippocampus, medial and lateral regions of striatum and prefrontal cortex. CREB signaling has been implicated in synaptic plasticity and the processing of memory (Silva, 2003). Numerous studies have demonstrated that CREB is one of the molecular signals in brain structures activated by the demands of a specific learning strategy (Colombo et al., 2003; Sung et al., 2008; Baudonnat et al., 2011). Furthermore, in tasks used to assess hippocampal involvement in learning and memory, such as the plus-maze task (which requires the processing of spatial information) and context fear conditioning, it has been shown that C57BL/6 mice perform better than DBA/2 mice (Paylor et al., 1994; Balogh et al., 2002; Passino et al., 2002). After spatial learning in the Morris water maze, levels of hippocampal pCREB of C57BL/6 mice were higher than those of DBA/2 mice, even though the two mouse strains did not differ in performance (Sung et al., 2008). In addition, levels of hippocampal pCREB are higher in rats that choose a place strategy on a plus maze task, whereas levels of striatal pCREB are higher in rats that choose a response strategy, compared with control rats (Colombo et al., 2003).

A number of studies have been conducted to reveal the neural structure or cellular mechanism responsible for flexibility in switching learning strategies in mice. These have reported that levels of acetylcholine release in the hippocampus increase and remain elevated throughout training when learning progressed sequentially from spatial to response strategies (Chang and Gold, 2003). In the present study, C57BL/6 mice performed better than DBA/2 mice at the learning strategy-switching task in Morris water maze (switching from cued to place learning or vice versa). Consistent with these behavioral differences, regardless of the learning strategy-switching order, hippocampal pCREB levels of C57BL/6 were higher than those of DBA/2. These results suggest that the hippocampus participates in this learning strategy-switching task, regardless of switching order.

The dorsal striatum also plays a role in cued learning (Colombo et al., 2003; Sung et al., 2008; Baudonnat et al., 2011). Studies have reported that the medial region is related to goal-directed learning, and the lateral region is related to response learning (Yin and Knowlton, 2006). Therefore, levels of pCREB in the medial and lateral regions of the dorsal striatum were measured upon completion of the learning strategy-switching task. Higher pCREB levels in the medial region of the dorsal striatum were observed in both strains of mice that received place-to-cued training than those that received training in the reverse order. No differences were observed in pCREB levels in the dorsolateral striatum between the two strains or two switching tasks. Several studies assert that the dorsomedial striatum is important for the spatial learning and reversal learning, but not the dorsolateral striatum (Pisa and Cyr, 1990; Devan and White, 1999). Also, the inactivation of the dorsomedial striatum showed deficit of shift learning between response and visual cue discrimination (Ragozzino et al., 2002). The anatomical linkage between dorsomedial striatum and prefrontal cortex in which brain area is related to behavioral flexibility is supposed by cortico-basal ganglia-thalamic loop (Groenewegen et al., 1990; Groenewegen and Berendse, 1994). Therefore, by our results, medial region of the dorsal striatum play a role in behavioral flexibility, in particular, in the swift from place to cued.

Animals with lesions to the prefrontal cortex show impairments in multiple switching tasks (Rich and Shapiro, 2007) or in reversal learning in the water maze (Boulougouris et al., 2007). The study recorded neuronal activity in the prefrontal cortex and also demonstrated selectivity in strategy switching (Rich and Shapiro, 2009). Anatomically, the prefrontal cortex makes efferent connections with the hippocampus via the thalamus (Vertes, 2002) and directly innervates striatum (Groenewegen et al., 1990). All evidence indicates that the prefrontal cortex, in addition to the hippocampus and dorsal striatum working in a competitive or cooperative way, is involved in the switching of learning strategies in the water maze (McDonald and White, 1994; Poldrack and Packard, 2003). In the present study, C57BL/6 mice exhibited superior performance to DBA/2 mice in the cued-to-place training switching trial. The behavioral superiority of C57BL/6 mice was accompanied by increased pCREB levels in prefrontal cortex relative to those in DBA/2 mice.

Unexpectedly, total CREB levels were also altered in relation to behavioral performance. CREB levels in prefrontal cortex were higher in C57BL/6 mice than in DBA/2 mice after the place-to-cued training. Furthermore, hippocampal CREB levels were increased in both strains of mice following cued-to-place training relative to those that received place-to-cued training. The study by Sung et al. (2008) also reported significant increases of total CREB in hippocampus after spatial training compared to cued training in these two inbred strains of mice. Additionally, it has been reported that levels of total CREB in hippocampus were lower in aged Fisher-344, Long Evans and Wistar rats with memory impairments compared to young adult rats (Brightwell et al., 2004; Trofimiuk et al., 2010; Morris and Gold, 2012). These results suggest that aged-related reductions in total CREB levels might contribute to aged-related memory impairment by the resultant reduction of pCREB levels (Morris and Gold, 2012). Likewise, increases of total CREB might be linked to increased phosphorylation levels in the prefrontal cortex and hippocampus by the specific demand of the learning strategy. However, further study is required to clarify the significance of increased CREB levels in response to cognitive demands.

In summary, the results of the present study provide new information about the behavioral performance of two inbred mouse strains that have common genetic backgrounds for transgenic and knock-out mice. Differences in their ability to switch learning strategies and in the expression levels of pCREB were detected between the strains, suggesting that these mice will be of interest for further study into the involvement of the hippocampus, striatum and prefrontal cortex in the implementation of different learning strategies. This alteration of pCREB expression is a dynamic process and the time course of the pCREB levels might be different according to the selected brain region and the learning strategy implemented by the task. Furthermore, the learning strategy-switching task used herein might be useful in studying cognitive function impaired by abundant intraneuronal Aβ accumulation and extracellular plague deposition that occurs in the brains of a transgenic mouse model of Alzheimer’s disease (Cho et al., 2014), which is used to study memory deficits and for the development of preventive treatments.

## Author Contributions

W-HC and J-SH conceived and designed the experiments. W-HC performed the experiments. W-HC and J-SH analyzed and discussed the data and wrote the manuscript.

## Conflict of Interest Statement

The authors declare that the research was conducted in the absence of any commercial or financial relationships that could be construed as a potential conflict of interest.
